# Miscellaneous

**Published:** 1874-06

**Authors:** 


### MISCELLANEOUS.

### A ROMANTIC TALE.

A FRAGMENT of a letter written by a lady at Naples to a friend in America, contains the following:

I mentioned that I got the particulars of a most romantic and interesting history lately, and I only now have found leisure to write them down for you. When in public with the Marchioness of S——, I had seen her frequently address a very pleasing, fine young woman, whose name and rank I knew, but nothing more; and she said she wished I should be better acquainted with her before she told me her history. She was reserved, but had a mild sort of quiet melancholy in her manner that attracted me very much, and you shall now learn the cause. I am not at liberty to give her full name, so you must be satisfied with her being called Rosalie, after her saint. She was the daughter of one of the first houses in this country, and brought into the world with every advantage, having been educated at home, and under a very amiable mother, who, unfortunately, died when she was only fifteen. Her father had selected a youth for her partner in life every way worthy of her; and, what seldom happens, the young people were allowed to form an attachment before marriage by a considerable degree of intimacy. The young count's mother was a high, violent character, but had not openly opposed this; however, she conducted herself in a manner that showed little partiality to her future daughter. All, however, went on well till a few days before the marriage. Great and splendid were the preparations, and future happiness appeared within their reach. The young people, as usual, were separated for the last two days. One hardly dare glance at the feelings with which they parted, to meet again in the happiest union—love and hope blinding them to all future chances against the completion of their happiness.

The evening before the marriage day Count P.'s mother came to his house, newly prepared for his bride, and said it had been resolved the marriage should take place on that night, privately, to spare his lovely Rosalie's feelings, as she shrunk from the public solemnity; and that all should be ready, and at an hour named he would be called for by the father. Accordingly, everything was so arranged; and the young man was conducted to church, his carriage following that of his supposed father-in-law. At the altar, which was dimly lighted, stood his mother and the bride, covered by a very thin silver-tissue veil, and the ceremony proceeded. The youth, whose thoughts were fixed on present happiness and engrossed by the service, distinguished no one, and received his wife in full confidence. Silent she was, but tranquil; and his mother carried her home. All the *cortege* parted, and he followed to his own house, there to unveil the treasure of his heart!

He found the saloon illuminated, and his brother and sister, who, on some pretence, had been kept absent from the ceremony, seemingly waiting in impatience with his mother and the bride. The doors closed after him, and his mother withdrew the veil, and discovered to him that his wife was a *beautiful idiot*, whose large estates she had long coveted, and had taken this most wicked manner of obtaining them for her family.

The anguish that followed brought him to the gates of death; and the loss of reason had nearly been the price at which she gained the success of a plan truly diabolical. His sister, a most amiable creature, soothed him at last into submission to his hard fate, after finding no means were left to set him free. Of the mother and the idiot I say nothing; he never saw either, I believe, from that hour.

Rosalie's fate, I believe, has drawn more tears than any event in real life ever did in Naples. Public proof was brought her father next morning of the marriage, but it was added that the bride, being veiled, her name was not known. Enraged, as you may conceive, he carried his daughter (in silence) to his villa, and there, I understand, with more tenderness than might have been expected from his stern character, unfolded what he deemed the treachery of her lover. The death blow to all her happiness was such, as her most interesting countenance proves, fifteen years cannot efface; and for a couple of years life seemed held by a very slender thread. That a young woman should remain unmarried out of a convent is a thing unknown; and her vast possessions made her father anxiously desire to see her married before the fatal truth was made known to her, as the sacredness of sorrow had kept aloof all intruders, and her father resolved she should return to the world under the protection of a husband.

How this was brought about may be accounted for by those who know the state of society here. All she desired, when she found that her father's will must be obeyed, was a full explanation of her situation to the Marquis of S——, whom she married.

Thus was the tragedy brought to the most trying scene—the discovery of her first lover's innocence, after she herself was another's. The marquis undertook this. He is a cold character, but to her appeared sincerely attached. Rosalie was now only nineteen when this hardest part of her trial was appointed to her; but the effects were quite different from what might have been looked for. The cup of misery appeared to have been overflowed, and she received the intelligence as a relief from the bitterness of her former pangs; and, grateful for his faith, she owned it was wisely done to place new duties before her ere she was acquainted with his share in their mutual misery.

Once, and only once, they met in private society; and she requested only her father and husband might be witnesses. With such a woman, what must have been the effect upon all present? She clasped him to her heart, and wept in his arms, and then turned to her husband, and said to Count P——: "To this generous man we owe this indulgence. Kneel with me, and swear it is the last intercourse we shall ever have together."

You may believe this noble woman's example won him to follow her upright views; and at no moment of their lives, during those years, has that vow ever been broken. In public they meet, but the life of each is exemplary. She fills the station of a wife and mother to perfection, and is rewarded by the respect of her husband and all her society. There is an elevated character in her sorrows and self-command that attracts my veneration; and as to him, I do think one of her most severe and secret pangs must be read in his faded form and fine, dejected countenance what he has suffered.

The idiot and mother both live, no one knows where. Count P—— married his sister to a Venetian, and devotes his time to her and her family.

### FRENCH SOCIAL CUSTOMS.

THE absence of the unmarried French woman in the American drawing rooms of Paris is the subject of general remark to transatlantic observers. There are American families of cultivation who have been living in Paris for ten years, and are not on terms of intimacy with a single French family, although they may have Frenchmen constantly at their tables. It is not the custom of the French to have an extensive social circle of friends, as in America; often it does not extend beyond their relations, among whom a praiseworthy harmony generally exists. There are many instances where Frenchmen have married Americans, but very few where Americans have married French women; but when it does occur, the doors of the interior are thrown open to them, and they are made acquainted with every feature of that private life hitherto closed to them.

These customs show the barriers which surround the interior life of the French people, and the difference which exists between them and us. However much the Americans may be disposed to adopt their customs, they are nowise inclined to adopt those of the Americans. One would think that when a marriage takes place between the Frenchman and the American girl, her intimate friends would have an opportunity of seeing something of the inner social life through the new connections thus created, but it is not generally the case. She is absorbed by her new relations, who have an aversion to that large circle of friends and acquaintances of which the Americans are usually so fond.—*Albert Rhodes, in The Galaxy for May.*

### TOLERANCE.

ABIMELECH came home on Sunday a little later than usual to dinner. That recalled to Mr. Slow's mind the fact that he had not seen his boy in the family pew during the reading of the "lethargy," and both facts suggested to him the bare possibility that he had not been at church.

"Abimelech," said Mr. Slow, solemnly, as he stood with his back toward the grate, "Abimelech, have you been to church?"

"Yes, sir," said Abimelech, stoutly; "I've been to the Universalist."

"Well, my son, I ain't like a good many fathers, that don't want their children to go anywhere but jest where they say. No, my son, I ain't none of these. Toleration is my motto—largest liberty, and all that—that our forefathers fit and bled for. Yes, my son, go where you please to meetin'. I don't care; only this I will say, that if I ketch you goin' to that meetin' again I'll take your hide off!"

### THE LAST MOMENTS OF A SINGLE GENTLEMAN.

This morning, April 1, at half-past eleven precisely, an unfortunate young man, Mr. Edward Pinkney, underwent the extreme penalty of infatuation, by expiating his attachment to Mary Anne Gale, in front of the altar railings of St. Mary's Church, Islington.

It will be in the recollection of those friends of the parties who were at the Jones' party at Bristol, two years ago, that Mr. Pinkney was there, and there introduced to Mary Anne, to whom he instantly began to direct particular attention—dancing with her no less than six sets that evening, and handing her things at supper in the most devoted manner. From that evening commenced the intimacy which terminated in this morning's catastrophe.

Poor Pinkney had barely attained to his twenty-eighth year, but there is reason to believe, that but for reasons of a pecuniary nature, his single life would have come earlier to an untimely end. A change for the better, however, having occurred in his circumstances, the young lady's friends were induced to sanction his addresses, and thus to become accessories to the course for which he has just suffered.

The unhappy man passed the last night of his bachelor existence in his solitary chamber. From half past eight to ten he was busily engaged in writing letters. Shortly after ten o'clock his younger brother, Henry, knocked at the door, when the doomed youth told him in a firm voice to come in. On being asked when he meant to go to bed, he replied, "Not yet." The question was then put to him how he thought he could sleep, to which his answer was, "I don't know." He then expressed a desire for a segar and a glass of grog, which were supplied him. His brother, who sat down and partook of the like refreshments, now demanded if he would want anything more that night. He said, "Nothing," in a firm voice. His affectionate brother then rose to take leave, when the devoted one considerately advised him to take care of himself.

Precisely at a quarter of a minute to seven the next morning the victim of Cupid, having been called according to his desire, rose and promptly dressed himself. He had the self-control to shave himself without the slightest injury, for not even a scratch upon his chin appeared after the operation. It would seem that he had devoted a longer time to his toilet than usual.

The wretched man was attired in a light blue dress coat, with frosted metal buttons, a white waistcoat, and nankeen trousers. He wore round his neck a variegated satin scarf, which partially concealed the Corazza of his bosom. In front of the scarf was inserted a breastpin of conspicuous dimensions. Having descended the staircase with a quick step, he entered the apartment where his brother and a few friends were awaiting him. He shook hands cordially with all present, and on being asked how he had slept, answered "Very well;" and to the further demand as to the state of his mind, he said, "I feel happy." One of the party having suggested that it would be as well to take something before the melancholy ceremony was gone through with, he exclaimed, with some emphasis, "Decidedly." Breakfast was accordingly served, when he ate the whole of a French roll, a large round of toast, two sausages, and three new laid eggs, which he washed down with two great breakfast cups of tea. In reply to an expression of astonishment on the part of a person present at his appetite, he declared that he never felt it better in his life.

Having inquired the time, and ascertained that it was ten minutes to eleven, he remarked that "It would soon be over." His brother then inquired if he could do anything for him, when he said he should like to have a glass of ale. Having drunk this he appeared satisfied.

The fatal moment now approaching, he devoted the remaining brief portion of his time to distributing among his friends those little articles which he would soon no longer want. To one he gave his segar case, to another his tobacco stopper, and he charged his brother Henry with his latch-key, with instructions to deliver it, after all was over, with due solemnity to his landlady.

The clock at length struck eleven, and at the same moment he was informed that a cab was at the door. He merely said, "I am ready," and allowed himself to be conducted to the vehicle, into which he got with his brother; his friends followed in others.

Arrived at the tragical spot a short but anxious delay of some seconds took place, after which they were joined by the lady, with her friends. Little was said on either side; but Miss Gale, with customary decorum, shed tears. Pinkney endeavored to preserve a composure, but a slight twitching in his mouth and eyebrows betrayed his inward agitation.

The ill starred bachelor having submitted quietly to have a large white bow pinned to his button-hole, now walked side by side with Miss Gale, with a firm step, to the altar. He surveyed the imposing preparations with calmness, and gazed unmoved on the clergyman, who, assisted by the clerk, was waiting behind the railings.

All requisite preliminaries having now been settled, the usual question was put, "Wilt thou have this woman for thy wife?" to which the rash youth replied, in a distinct voice, "I will." He then put the fatal ring on Miss Gale's finger, the hymenial noose was adjusted, and the poor fellow was launched into—matrimony.—*Punch.*

### COLOGNE.

Cologne water was originally a quack medicine, described as an "elixir which has the power of restoring the parts of the body that are attacked by any disease or predisposition to the same, of fortifying and reëstablishing their natural functions, or insinuating into them a moderate and living warmth, which, sympathizing with their own, reanimates their vital forces," etc. A correspondent of the Boston *Medical and Surgical Journal* gives a copy of an advertising circular, dated January 13, 1827, which, after describing the effects of eau de cologne as above, warns the public against purchasing the elixir from any one but Antoine Farine, of Cologne, to whom the secret of its concoction had been revealed by Signor Paul Feminis, the inventor, who was a native of the same city. It cannot be expected, therefore, that cologne water purchased from any one else will produce the effects promised for the great orginal.

### A RELIC OF THE VIKINGS.

THE oaken hull of a vessel, supposed to date from the time of the old Vikings of the North, was recently discovered, while digging a tumulus near Frederickstadt, in Norway. It was rather flat and low in the water, tapering to points at each end, with a length of keel of 44 feet and a breadth of beam of 13 feet. It is supposed to have been used as a war vessel for coast service, being propelled by oars and sails. An ancient practice in Norway was to place the vessel over the remains of its captain; and fragments of dress, horse accoutrements and harness have been discovered under this. This is deemed quite a prize for the archæologists, and the entire lot is to be placed in the Antiquarian Museum at Christiana.

### A CURIOUS UNIFORMITY.

ONE of the most wonderful things in the world is the uniformity of apparent accidents. Thus, in Great Britain, where the statistics are much better preserved than in this country, the total number of deaths by human violence in 1870 was 16,953; in 1869 it was 16,497; it was 16,968 in 1868; 16,866 in 1867;

16,915 in 1866; 17,374 in 1865, and 17,018 in 1864. Not only that, but the same uniformity extends to the manner in which the victims met their deaths. How are we to account for this uniformity, except upon the principle that in the divine economy of Nature there is no such thing as an accident or an exception to ordinary laws by which the whole universe is governed?

### GOOD LANGUAGE.

YOUNG people should acquire the habit of correct speaking and writing, and abandon as early as possible any use of slang words and phrases. The longer you put this off the more difficult the acquirement of correct language will be; and if the golden age of youth, the proper season for the acquisition of language, be passed in its abuse, the unfortunate victim will most probably be doomed to talk slang for life. You have merely to use the language which you read, instead of the slang which you hear, to form a taste in agreement with the best speakers and poets in the country.

### LOVE AND PRIDE.

"MY life is like the clouds," she said,
"So sombre, leaden, gray—
Ere warmed by sunset's crimson glow
It slowly fades away."

"My life is like the flower," she said,
"Nipped by untimely frost,
For o'er my youth's glad opening day
A chilling shade has crossed."

"My life was like a bird's," she said,
"Wooed by a gentle hand,
Till from my heart love's blinding sway
Was freed at pride's command."

"Pride's torch is fallen now," she said,
"And quenched its lurid light;
But will my dream of love return
From the dark realms of night?"

### HOW THE INDIANS DO IT.

THE *Hartford Courant* evolves the following theory: "There has been some philological doubt as to the origin of the phrase 'After him with a sharp stick.' It may have occurred to many that the 'sharp stick' referred to is the much feared 'January bill.' And it would seem that there is some ground for this. The Neeshenan Indians of California have not the brutal and disagreeable habit prevalent among us of sending dunning bills. When one Indian owes another, it is considered bad taste, as it is, for the creditor to dun the debtor. He proceeds with more delicacy. He procures a certain number of little sticks, according to the amount of the debt, and paints a ring around the end of each. These he carries and tosses into the debtor's wigwam and then goes away without a word. The debtor pays the debt and destroys the sticks; it is considered a reproach to have these dunning sticks thrown into the wigwam, and the creditor never uses them except with hard customers."

### JOY.

O JOY, hast thou a shape?
  Hast thou a breath?
How fillest thou the soundless air?
Tell me the pillars of thy house!
What rest thee on? Do they escape
  The victory of death?
And are they fair
  Eternally, who enter in thy house?
O Joy, thou viewless spirit, cans't thou dare
  To tell the pillars of thy house?
On adamant of pain,
  Before the earth
Was born of sea, before the sea,
Yea, and before the light, my house
Was built. None know what loss, what gain
  Attends each travail birth.
No soul could be
  At peace when it had entered in my house,
If the foundations it could touch or see
  Which stay the pillars of my house!

### AN EDITOR WHO IS DEAF.

WE thought everybody in the State knew that we are deaf, but once in awhile we find one who is not aware of the fact. A female book peddlar came to the office the other day. She wished to dispose of a book. She was alone in the world, and had no one to whom she could turn for sympathy or assistance, hence we should buy her book. She was unmarried, and had no manly heart into which she could pour her sufferings, therefore we ought to invest in her book. She had received a liberal education, and could talk French like a native, we could not, in consequence, pay her less than $2 for a book. We had listened attentively, and here broke in with, "What do you say? We're deaf." She started in a loud voice and went through her rigmarole. When she had finished, we went and got a roll of paper and made it into a speaking trumpet, placed one end to our ear, and told her to proceed. She nearly broke a blood vessel in her effort to make herself heard. She commenced: "I am alone in this world——" "It doesn't make the slightest difference to us. We are a husband and father. Bigamy is not allowed in this State. We are not eligible to proposals." "Oh, what a fool the man is," she said, in a low tone; then at the top of her voice, "I don't want to marry you, I want to sell a b-o-o-k." This last sentence was howled. "We don't want a cook," we remarked blandly; "our wife does the cooking, and she wouldn't allow as good looking a woman as you to stay in the house five minutes. She is very jealous." She looked at us in despair. Gathering her robes about her, giving us a glance of contempt, she exclaimed: "I do believe that if a 300-pounder were let off alongside that deaf fool's head he'd think somebody was knocking at the door." You should have heard her slam the door when she went out. We heard that. —*Santa Clara* (Cal.) *Echo.*

PETS should always be tolerated, for they have their proper place in every household. If they furnish to the young imaginary playfellows, if they help older people to forget the cares of the present, and soften in them the austerities of this hard world's life—if, above all, they can be made morally significant, let us not condemn them as unworthy of our regard.

### HEALTH RESORTS.

(*Continued from the May number.*)

### WATKINS GLEN.

"Glen Cathedral" is a term that seems to have adhered with some tenacity, but the other waterfalls and pools have almost as many terms as there are different tastes and fancies among the visitors; and names, at best, apply to one feature only of the scene they describe, whereas in each picture there are usually a hundred phases that rival each other in beauty and interest. In this strange rift in the rocks the eye shifts from beauty to beauty, from marvel to marvel, with restless delight.

The tumbling waterfalls; the dark, silent pools; the light above reflecting from cliff to cliff, and glancing with rich beauty on rock and cascade; the fantastic growths of trees at every "point of vantage" and the interlacing branches above; the picturesque bridges and stairways; the profound silence, broken only by the sound of waters—all these conditions make up a fascinating charm that each succeeding picture varies in detail, but which pertain with almost equal force to every part of the entire glen.

Among the strange beauties of the place are the dark pools that lie at the foot of the cascades. The water in this strange gorge is of a brilliant green and beautifully transparent. In shallow places it is of an almost perfect emerald hue, and in deep pools becomes the darkest sea green. There is one pool which no one has ever been able to fathom. A pole thirty feet long thrust into it totally disappears, never returning to the surface. It is assumed that a channel exists far down under the rock, the subterranean current thus created sweeping objects let down in the pool out of reach or of power to return. The cliffs at the deepest part of the gorge have an elevation of about three hundred feet.

### MAGNETIC AND SULPHUR SPRINGS.

Opposite the entrance to the Watkins Glen, and connected with the Glen Park Hotel, are springs which have attained considerable reputation for the cure of nervous diseases and various chronic affections. The Glen Park Hotel is one of the best appointed and most comfortable hotels at Watkins.

### HAVANA GLEN.

Three miles south of Watkins is "Havana Glen." It is very picturesque, more airy, and is quite as easy of access, but is wanting in those elements of gloom, and vastness, and solemn grandeur which are the peculiar characteristics of Watkins Glen. Nevertheless, there is a class of tourists who admire Havana Glen even more than its great rival. There are many cascades. The same system of stairways and ladders prevails as at Watkins, but these aids to progress are fewer in the former and the paths broader. The Glen, moreover, is short, as compared with Watkins, while the height, from the level of the valley to the table land above, is much less. In the early summer months the water is greater than at Watkins, but it is said to shrink almost to a thread during the heats of July and August, while that of Watkins, being fed from cold springs far up the mountain, is much more permanent, though subject to the influence of the seasons.

### MAGNETIC SPRINGS.

These flowing springs are situated between the Havana and Watkins Glens. They are connected with the "Magnetic Springs Sanitarium," a home for tourists, pleasure seekers and invalids. The magnetic property of these waters was discovered only two or three years since, but many remarkable cures of rheumatism, diseases of the kidneys and other chronic troubles are reported. The Sanitarium is in charge of a thoroughly competent physician.

EDITORIAL NOTICE.—HECKERS' FARINA is well known as one of the most economical and nutritious preparations ever brought to the table; when prepared as a jelly it is very palatable and at the same time is a beautiful ornament; in the form of a gruel, as a food for the sick and for children, it is unequalled. HECKERS' Cracked Wheat, as an article of food, is not excelled by any other preparations of wheat for the specific design of producing and maintaining a healthful, active condition of the organs of digestion, assimilation, etc. To those of sedentary habits the use of wheat prepared in this form cannot be too highly estimated; and where it has been used by such persons, its virtues have made it a favorite article of daily food, in some shape or other. Its effect on the stomach and alimentary canal is soothing; and while imparting a high degree of nutrition, it promotes a healthy action of the viscera, similar to that induced by judicious exercise—a result sought for in vain from cathartic medicines, which leave the internal system more or less irritated, or weakened, and often seriously deranged. Flour and the other articles are put up for the retail trade in convenient packages, so that families may enjoy the luxury of continual freshness, an essential point in almost every vegetable preparation destined for human sustenance.

